# The Influence of Renal Function Impairment on Kappa Free Light Chains in Cerebrospinal Fluid

**DOI:** 10.1177/11795735211042166

**Published:** 2021-11-19

**Authors:** Franz F Konen, Philipp Schwenkenbecher, Ulrich Wurster, Konstantin F Jendretzky, Nora Möhn, Stefan Gingele, Kurt-Wolfram Sühs, Malte J Hannich, Matthias Grothe, Torsten Witte, Martin Stangel, Marie Süße, Thomas Skripuletz

**Affiliations:** 1 Department of Neurology, 196170 Hannover Medical School, Hannover, Germany; 2 Institute of Clinical Chemistry and Laboratory Medicine, 560776 University Medicine Greifswald, Greifswald, Germany; 3 Department of Neurology, 197718 University Medicine Greifswald, Greifswald, Germany; 4 Department of Clinical Immunology & Rheumatology, Hannover Medical School, Hannover, Germany

**Keywords:** cerebrospinal fluid, reiber’s diagram, kappa free light chains (KFLC), renal function, eGFR, biomarker

## Abstract

**Background:**

The determination of kappa free light chains (KFLC) in cerebrospinal fluid (CSF) is an upcoming biomarker for the detection of an intrathecal immunoglobulin synthesis. Since renal function impairment leads to altered serum KFLC and albumin concentrations, interpretation of KFLC in CSF may be influenced by these parameters.

**Methods:**

In this two-center study, the influence of renal function (according to the CKD-EPI creatinine equation) on KFLC and albumin concentrations was investigated in patients with “physiological” (n = 139), “non-inflammatory” (n = 146), and “inflammatory” (n = 172) CSF profiles in respect to the KFLC index and the evaluation in quotient diagrams in reference to the hyperbolic reference range (KFLC IF).

**Results:**

All sample groups displayed declining KFLC indices and KFLC IF values with decreasing renal function (*P*-values between <.0001 and .0209). In “inflammatory” CSF profile samples, 15% of the patients presented a KFLC index <5.9 while 10% showed an intrathecal KFLC fraction below Q_Kappa_(lim), suggesting possible false negative KFLC results.

**Conclusions:**

The influence of renal function should be considered while interpreting KFLC results in patients with neuroinflammatory diseases. The interpretation of KFLC in quotient diagrams is less susceptible to renal function impairment than the KFLC index and should be preferentially used.

## Introduction

Oligoclonal bands are the gold standard for detection of intrathecal immunoglobulin G (IgG) synthesis in neuroinflammatory diseases and are part of the latest revision of the McDonald criteria to diagnose multiple sclerosis (MS) of 2017.^1,2^ Applying these criteria, MS can be diagnosed more frequently at the time of the first clinical event due to the implementation of oligoclonal bands as a substitute for dissemination in time.^3^ Different studies have shown that measurement of kappa free light chains (KFLC), which are synthesized in excess during the assembly of immunoglobulins, might be an alternative method to show an immunoglobulin synthesis in the central nervous system (CNS).^4–7^ In the past years, the quotient of KFLC in cerebrospinal fluid (CSF) and serum (Q_KFLC_) with reference to the albumin quotient (Q_Alb_), the so-called KFLC index, represented the most common method to interpret intrathecal KFLC concentrations, despite missing consensus about the empirically defined threshold value.^5–9^ In 2019, Reiber and colleagues presented an empirically derived, Q_Alb_-dependent hyperbolic function in analogy to the already established quotient diagrams for the immunoglobulins G, A, M.^10,11^ In recent studies, Reiber’s hyperbolic threshold-line for the Q_KFLC_ showed a higher sensitivity in the diagnosis of MS compared with other non-linear functions.^7,12^ However, since serum KFLC is excreted with urine, renal function is supposed to be a crucial influence factor, which should be considered. Serum KFLC has a half-life time of 2-6 hours *in vivo* due to renal elimination and accumulates when renal function is impaired.^13^ A recent report pointed out that in three cases with positive oligoclonal bands but negative KFLC index (<3.6), considerably increased (>50 mg/l) serum values of KFLC were observed. The authors advised caution that the sensitivity of the KFLC index depends on the KFLC serum concentration.^6^ Further, albumin concentrations in serum are lowered by renal function decline, thus affecting the KFLC index as well as the KFLC interpretation in quotient diagrams based on Q_Alb_.^14^ However, systematic investigations of the impact of a decreased renal function on the concentration of KFLC in CSF and serum are lacking. Thus, the aim of this study was to systematically analyze the effects of renal function on KFLC and albumin concentrations and the calculated intrathecal synthesis of KFLC by using the most common KFLC index of 5.9 and the KFLC quotient diagram proposed by Reiber and colleagues.^9,11^

## Methods

### Patients

This two-center study compromises a total of 457 patients, who presented at the Department of Neurology of the Hannover Medical School (MHH) or the Department of Neurology of the University Medicine Greifswald (UMG) between 2008 and 2019. The pairs of CSF and serum used for this study were collected as part of routine diagnostics. Some of these patient samples were previously investigated with focus on different aspects.^4,6,7,15,16^ The included samples were divided in three cohorts according to the CSF basic profile: (1) “physiological” CSF profile (n = 139), defined as CSF cell count <5/μl, total protein <500 mg/l, age-adjusted CSF lactate concentration <2.1 mmol/l for 16-50 years; <2.6 mmol/l for over 51 years and normal age-adjusted Q_Alb_ values (<4 + (age in years/15)*10^−3^), no evidence of intrathecal immunoglobulin synthesis in quotient diagrams and oligoclonal band analysis.^10,17,18^ (2) “Non-inflammatory” CSF profile (n = 146), defined as absence of pleocytosis (CSF cell count <5/μl) and no evidence of an intrathecal immunoglobulin production interpreted with quotient diagrams or oligoclonal band analysis.^10,18^ In contrast to the cohort with “physiological” CSF profile, patient samples of this group did not entirely fulfill the “physiologic” CSF parameters as total protein concentration, Q_Alb_, or lactate concentration could have revealed pathologic results. (3) “Inflammatory” CSF profile (n = 172), defined as patient samples with detection of oligoclonal bands in CSF. Patient samples with pleocytosis but without detection of oligoclonal bands were not included. Further demographic data and CSF parameters of the included patient samples are shown in Table 1 and Supplementary Material.

**Table 1.** Demographic data and cerebrospinal fluid (CSF) parameters. Included are patient’s characteristics, basic CSF, and kappa free light chain (KFLC) findings as well as renal function estimated by glomerular filtration rate (eGFR) (ml/min/1.73 m²) according to CKD-EPI equation. Three groups were included: samples of patients with “physiological” CSF profile, “non-inflammatory” CSF profile, and “inflammatory” CSF profile.

	Physiological CSF Profile (n = 139)	Non-inflammatory CSF Profile (n = 146)	Inflammatory CSF Profile (n = 172)
Age in years, mean (min–max)	49 (18–89)	58 (18–87)	38 (18–83)
Females, n (%)	61/139 (44%)	74/146 (51%)	111/172 (65%)
Renal function estimated by eGFR (ml/min/1.73 m^2^) according to CKD-EPI equation, mean (min–max)	93 (5–164)	82 (9–134)	104 (47–143)
Serum albumin concentration (g/l), mean (min–max)	44.2 (30.9–57.6)	41.1 (15.5–51.3)	41.9 (11.6–54.1)
CSF albumin concentration (mg/l), mean (min–max)	212.4 (93.4–347)	390 (94.7–1550)	248.6 (64.9–1070)
Serum KFLC concentration (mg/l), mean (min–max)	15 (5.6–90.5)	18.1 (7–130)	11.8 (.64–43.2)
CSF KFLC concentration (mg/l), mean (min–max)	.15 (.03–.7)	.28 (.033–1.9)	4.5 (.09–35.2)
Reiber’s diagram for KFLC positive (>Q_Kappa_(lim)), n (%)	7/139 (5%)	8/146 (5%)	155/172 (90%)
Intrathecal KFLC fraction in relation to Q_mean_ >0% (Reiber’s diagram), n (%)	74/139 (53%)	59/146 (40%)	167/172 (97%)
CSF cell count (>4/μl CSF), n (%)	0/139	0/146	101/172 (59%)
CSF total protein (>500 mg/l), n (%)	0/139	97/146 (66%)	42/172 (24%)
Q albumin (>4 + (age in years/15)*10^−3^), n (%)	0/139	81/146 (55%)	49/172 (28%)
CSF lactate concentration (age-adjusted >2.1 mmol/l (16-50 years), >2.6 mmol/l (>51 years), n (%)	0/139	31/146 (21%)	46/172 (27%)
Intrathecal IgG synthesis (>0%), n (%)	0/139	0/146	91/172 (53%)
Intrathecal IgA synthesis (>0%), n (%)	0/139	0/146	18/172 (10%)
Intrathecal IgM synthesis (>0%), n (%)	0/139	0/146	56/172 (33%)
Oligoclonal bands in CSF, n (%)	0/139	0/146	172/172 (100%)

The investigation was approved by the Ethics Committee of MHH (No. 7837_BO_K_2018, April 6, 2018) and UMG (Votum III UV 39/03, May 22, 2003). This is a retrospective study, and only data were included that were evaluated for patients treatment or diagnostic purposes as part of the clinical routine (routine CSF analytics as described below; routine blood laboratory analysis including blood panel, coagulation analysis, parameters for renal and hepatic function). Thus, the local ethics committee waived the need for written informed consent from the participants. The data used in this study was anonymized before its use.

### Analytical Procedures

All paired CSF and serum samples were analyzed according to routine diagnostic procedures in the Neurochemistry Laboratory of the Department of Neurology of the MHH and the Interdisciplinary CSF Laboratory of the UMG. Kinetic nephelometry (Beckman Coulter IMMAGE, Brea, CA, USA (MHH); BN ProSpec, Siemens Healthcare Diagnostics Products GmbH, Marburg, Germany (UMG)) was used to measure concentrations of albumin, IgG, IgM, and IgA in serum and CSF samples. Intrathecal synthesis of IgG, IgA, and IgM was calculated in quotient diagrams.^10^ CSF oligoclonal bands were detected by isoelectric focusing in polyacrylamide gels (EDC, Tübingen, Germany) with consecutive silver staining (MHH) or using isoelectric focusing with a semiautomatic agarose electrophoresis system (Hydragel 9 CSF, Hydrasys 2 Scan, Sebia GmbH, Fulda, Germany) (UMG).^19^ The concentration of KFLC in CSF and serum samples was determined by nephelometry with the N Latex FLC kappa Kit (Siemens Healthcare Diagnostics Products GmbH, Erlangen, Germany) according to the manufacturer’s instruction on a BN Prospec analyzer. CSF pre-dilution was set to 1:2 and serum pre-dilution was set to 1:100. The lower limit of quantification of the assay was .034 mg/l. The hyperbolic reference range as well as the amount of intrathecally synthesized KFLC in relation to Q_mean_ was calculated according to the formulas described by Reiber et al (discrimination line: Q_Kappa_ (lim) = (3.27 (Q_Alb_^2^ + 33)^0.5^−8.2) ×10^−3^; reference range: Q_Kappa_ (mean) ± 3 CV).^11^ The estimated glomerular filtration rate (eGFR) was calculated by the CKD-EPI creatinine equation.^20^

### Statistical Analysis

Statistical analysis was performed with GraphPad Prism (La Jolla, CA, USA; version 5.02). The level of statistical significance was set to 5%. The D’Agostino & Pearson omnibus normality test was used to assess for normal distribution of values. Data were described as minimum, maximum (min-max), and mean. Mann–Whitney U-test was used to analyze independent values. Kruskal–Wallis test and Friedman test with Dunn’s Multiple Comparison posthoc test were used for group comparison. To examine for a significant correlation, Spearman’s r (Gaussian distributed values) and Pearson’s r (nonparametric distributed values) were used and *P*-values as well as the coefficient of correlation (ρ) were depicted.

## Results

### Patient Samples With “Physiological” CSF Profile

**Figure 1.**
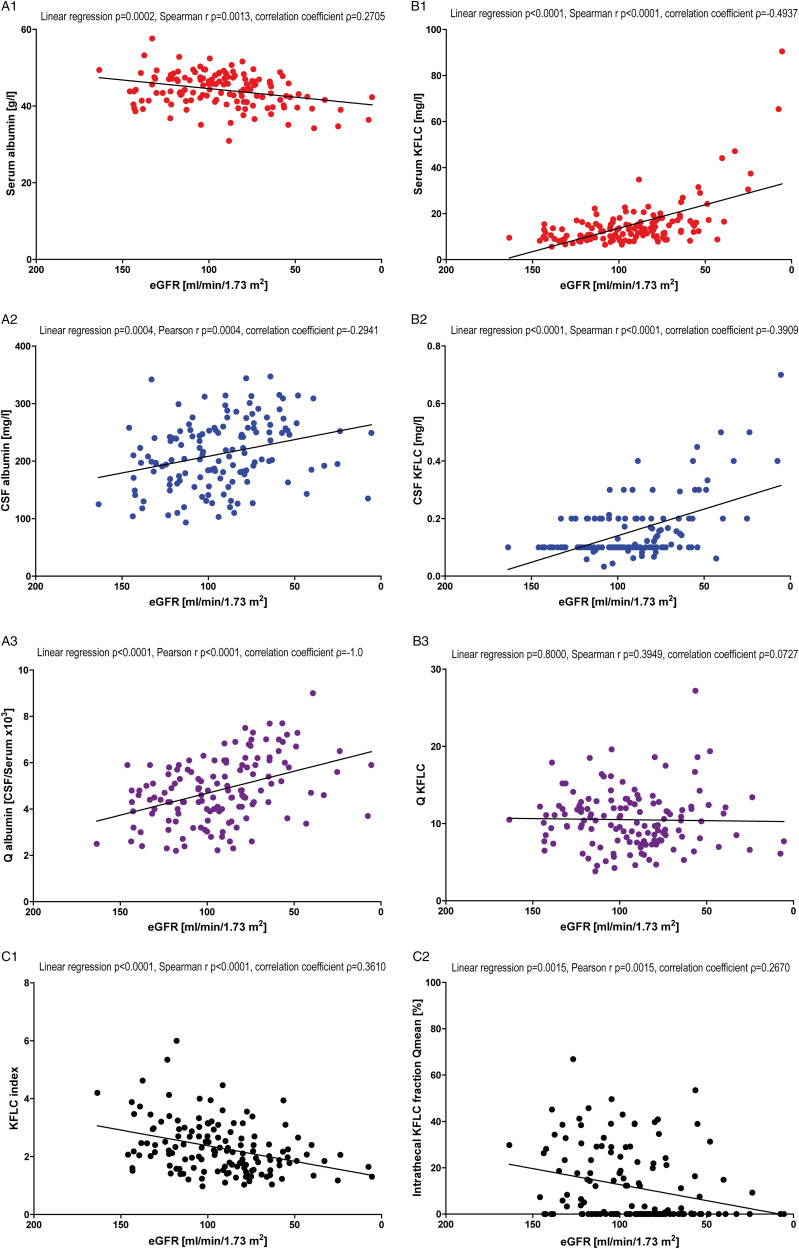
Correlation with renal function in “physiological” cerebrospinal fluid (CSF) profile patients. Depicted are the correlations of serum albumin (A1), cerebrospinal fluid (CSF) albumin (A2), serum kappa free light chain (KFLC) (B1), and CSF KFLC (B2) concentrations with renal function estimated by the glomerular filtration rate (eGFR) according to the CKD-EPI equation in “physiological” CSF profile patients. Further, the correlation between CSF/serum quotients of albumin (A3) and KFLC (B3) and eGFR are shown. In C1, the correlation between eGFR and KFLC index (Q_KFLC _/ Q_Alb_) is depicted, while C2 presents the correlation between eGFR and the intrathecal KFLC fraction in relation to Q_mean_ according to Reiber’s diagram for KFLC (KFLC IF). In the caption, *P*-values of linear regression and Spearman’s r (Gaussian distributed values) or Pearson’s r (nonparametric distributed values) as well as the coefficient of correlation (ρ) are shown.

This cohort included 139 patient samples with “physiologic” CSF parameters as shown in Table 1 and Figure 1. It was observed that albumin concentrations decreased in serum with declining renal function (*P* = .0002, Figure 1, A1), while CSF albumin values increased (*P* = .0004, Figure 1, A2). As a consequence, calculating the albumin ratios (albumin in CSF/albumin in serum) revealed significantly higher Q_Alb_ values with declining renal function (P < .0001, Figure 1, A3).

In contrast to albumin, both serum and CSF KFLC concentrations increased significantly and revealed a linear correlation with declining renal function (both P < .0001, Figure 1, B1 and B2). The quotient of CSF KFLC and serum KFLC concentrations remained unaffected of the eGFR and showed neither a linear connection (*P* = .8) nor a significant correlation (*P* = .3949, Figure 1, B3).

In concordance to the unaffected Q_KFLC_ and the rising Q_Alb_, the KFLC index (Q_KFLC_/Q_Alb_) declined and a significant correlation was observed with decreasing renal function (*P* < .0001, Figure 1, C1). The intrathecal KFLC fraction in relation to Q_mean_ according to Reiber’s diagram for KFLC (KFLC IF) tended to lower levels with declining renal function and a significant correlation between eGFR and KFLC IF was found (*P* = .0015, Figure 1, C2).

### Patient Samples With “Non-inflammatory” CSF Profile

**Figure 2.**
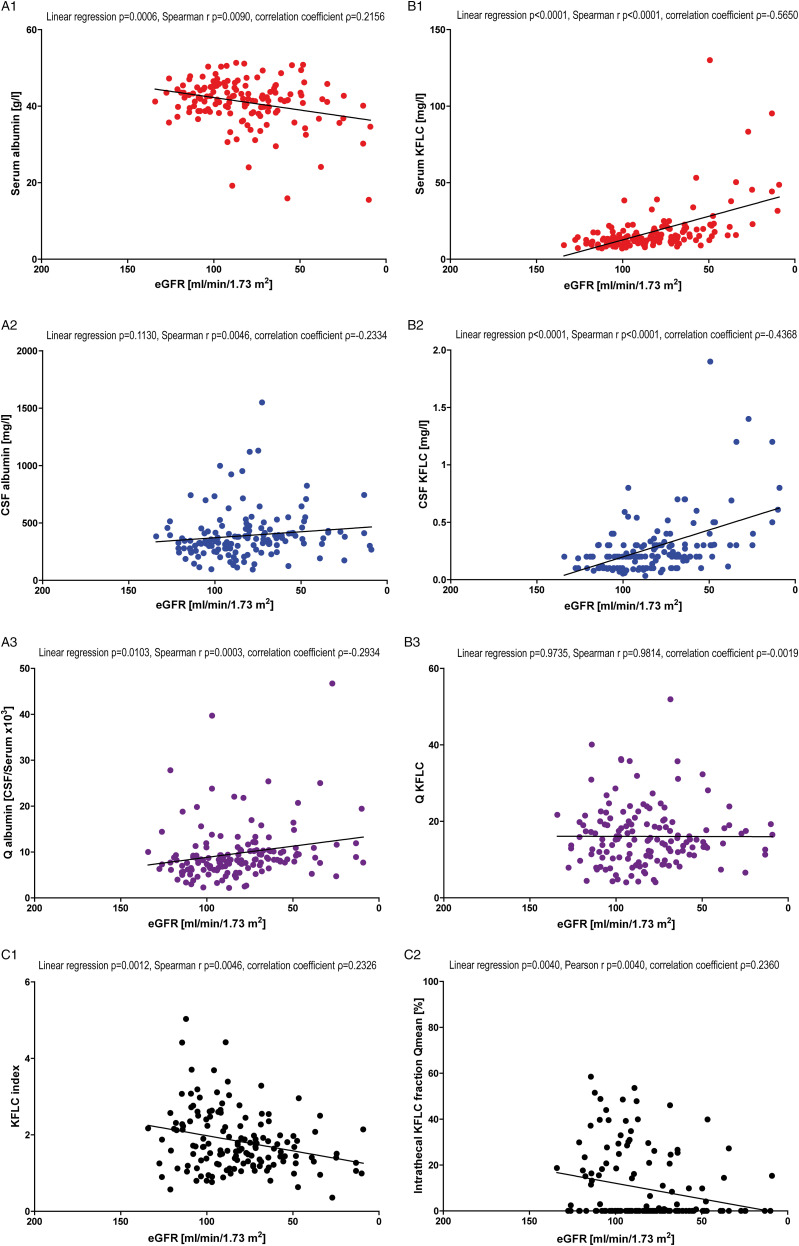
Correlation with renal function in “non-inflammatory” cerebrospinal fluid (CSF) profile patients. Depicted are the correlations of serum albumin (A1), cerebrospinal fluid (CSF) albumin (A2), serum kappa free light chain (KFLC) (B1), and CSF KFLC (B2) concentrations with renal function estimated by the glomerular filtration rate (eGFR) according to the CKD-EPI equation in “non-inflammatory” CSF profile patients. Further, the correlation between CSF/serum quotients of albumin (A3) and KFLC (B3) and eGFR is shown. In C1, the correlation between eGFR and KFLC index (Q_KFLC_/Q_Alb_) is depicted, while C2 presents the correlation between eGFR and the intrathecal KFLC fraction in relation to Q_mean_ according to Reiber’s diagram for KFLC (KFLC IF). In the caption, *P*-values of linear regression and Spearman’s r (Gaussian distributed values) or Pearson’s r (nonparametric distributed values) as well as the coefficient of correlation (ρ) are shown.

This cohort included 146 patients with “physiologic” CSF parameters except for Q_Alb_, CSF lactate concentration and CSF total protein and no evidence for an intrathecal immunoglobulin synthesis as shown in Table 1 and Figure 2. Albumin concentrations significantly decreased in serum with declining renal function (*P* = .0006, Figure 2, A1) while CSF albumin values showed a tendency to a negative association even though the examined association was not statistically significant (*P* = .1130, Figure 2, A2). Calculating the albumin quotients (albumin in CSF/albumin in serum) revealed slightly higher Q_Alb_ values with declining renal function (*P* = .0103; Figure 2, A3).

Serum and CSF KFLC concentrations increased significantly and revealed a linear correlation with decline of renal function (both P < .0001, Figure 2, B1 and B2). The Q_KFLC_ remained unaffected of the eGFR and neither a linear connection (*P* = .9735, Figure 2, B3) nor a significant correlation (*P* = .9814, Figure 2, B3) was observed.

The unaffected KFLC quotient and rising albumin quotient resulted in a significant decline of the KFLC index (Q_KFLC_/Q_Alb_) with decreasing renal function (*P* = .0012, Figure 2, C1). Furthermore, a significant correlation between declining renal function and decrease of KFLC IF was observed (*P* = .0040, Figure 2, C2).

### Patient Samples With “Inflammatory” CSF Profile

**Figure 3.**
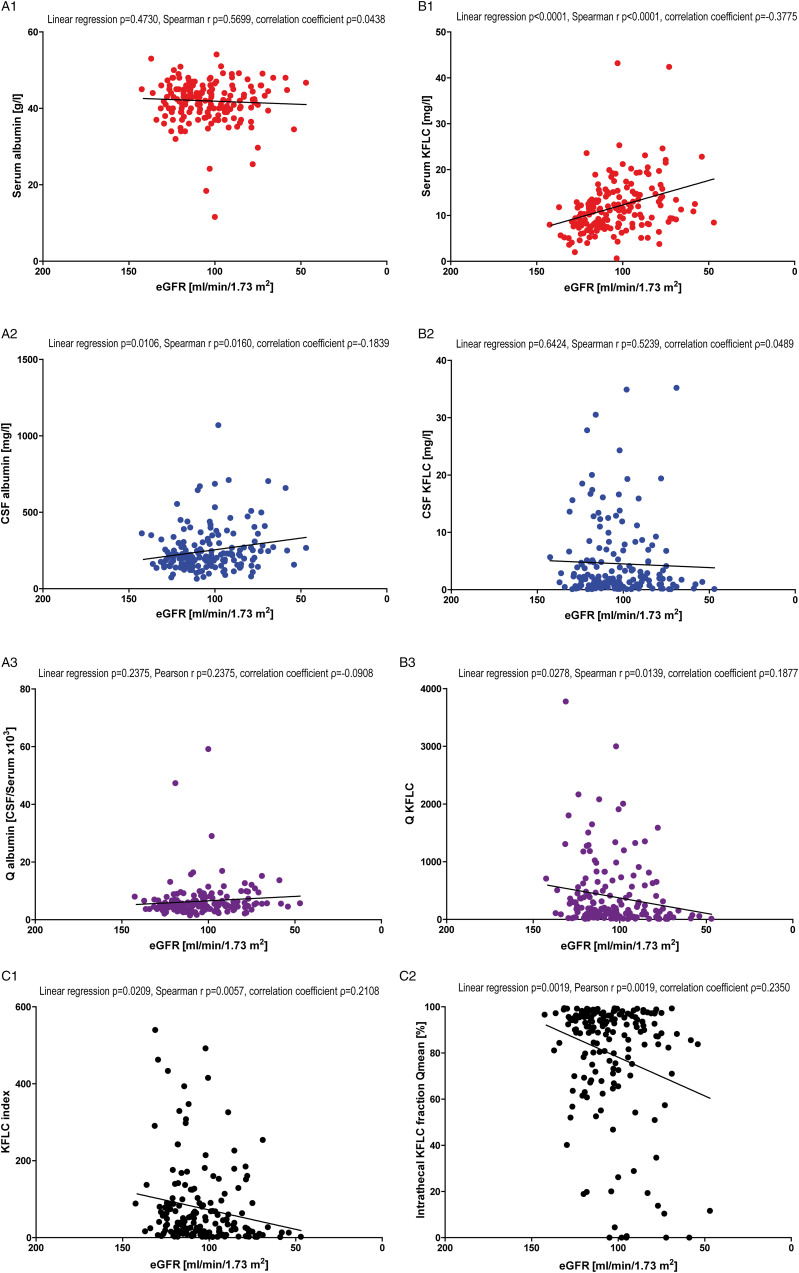
Correlation with renal function in “inflammatory” cerebrospinal fluid (CSF) profile patients. Depicted are the correlations of serum albumin (A1), cerebrospinal fluid (CSF) albumin (A2), serum kappa free light chain (KFLC) (B1), and CSF KFLC (B2) concentrations with renal function estimated by the glomerular filtration rate (eGFR) according to the CKD-EPI equation in “inflammatory” CSF profile patients. Further, the correlation between CSF/serum quotients of albumin (A3) and KFLC (B3) and eGFR is shown. In C1, the correlation between GFR and KFLC index (Q_KFLC_/Q_Alb_) is depicted, while C2 presents the correlation between eGFR and the intrathecal KFLC fraction in relation to Q_mean_ according to Reiber’s diagram for KFLC (KFLC IF). In the caption, *P*-values of linear regression and Spearman’s r (Gaussian distributed values) or Pearson’s r (nonparametric distributed values) as well as the coefficient of correlation (ρ) are shown.

The “inflammatory” CSF profile patient samples included 172 patients all of whom displayed oligoclonal bands in CSF (Table 1 and Figure 3). In this group, albumin concentrations in serum did not correlate significantly with declining renal function (*P* = .4730, Figure 3, A1), while CSF albumin values increased significantly with declining renal function (*P* = .0106, Figure 3, A2). Q_Alb_ did not show significant results with decline in renal function (*P* = .2375; Figure 3, A3).

While serum KFLC concentrations increased significantly with declining renal function (P < .0001, Figure 3, B1), CSF KFLC concentrations remained unchanged (*P* = .6424, Figure 3, B2). The Q_KFLC_ decreased significantly with reduced renal function (linear regression *P* = .0278, Spearman’s r *P* = .0139, Figure 3, B3).

In concordance to the decreasing KFLC ratios and unaffected albumin ratios, the KFLC index (Q_KFLC_/Q_Alb_) declined and a significant correlation was observed with decreasing renal function (*P* = .0209, Figure 3, C1). KFLC IF as well declined significantly with reduction of renal function (*P* = .0019, Figure 3, C2).

A KFLC index below the most commonly used cut-off value of 5.9 was found in 25/172 “inflammatory” patient samples.^9^ 2 of the 25 patients presented a reduced eGFR below 60 ml/min/1.73 m^2^. By applying the Reiber’s diagram for KFLC, 17/172 patient samples showed an intrathecal KFLC fraction below Q_Kappa_(lim). 2 of the 17 patients presented a reduced eGFR. The eGFR of the patients with Reiber’s diagram for KFLC negativity varied between 47 and 120 ml/min/1.73 m^2^ (mean 89 ml/min/1.73 m^2^) and was significantly lower compared with the mean eGFR of the whole “inflammatory” CSF profile patient group (104 ml/min/1.73 m^2^; *P* = .0036). Of the “inflammatory” patient samples with a KFLC index below 5.9 or Reiber’s diagram for KFLC negativity, 40% respective 35% suffered from autoimmune mediated or infectious processes of the CNS (according to diagnoses in Supplementary Material).

### Age-dependent Correlations

As renal function impairment is more frequent in aged patients, we further performed analyses of possible age associated influences on serum and CSF proteins. The investigation of the influence of patient’s age on CSF and serum albumin as well as KFLC concentrations revealed similar results as for the correlation with eGFR (Supplementary Material).

In all patient sample groups, a significant correlation between rising age and declining renal function was observed (P < .0001, Supplementary Material). All samples revealed a significant decrease of serum albumin concentrations with rising age, while CSF albumin concentrations increased, consecutively leading to a significant elevation of Q_Alb_ in samples of older patients (*P*-values between < .0001 and .0002, Supplementary Material). KFLC concentrations in serum and CSF increased with increasing patient age over all patient sample groups thus a significant linear regression or correlation was not observed for Q_KFLC_ (*P*-values between .0623 and .7551, Supplementary Material).

In concordance to unaltered Q_KFLC_ and rising Q_Alb_ with increasing age, a significant decrease of the KFLC index with increasing age was observed (*P*-values between .0027 and .0475, Supplementary Material). In contrast to eGFR-based correlations, a significant decrease of KFLC IF with increasing patient age was not observed in any patient sample group (Supplementary Material).

**Figure 4.**
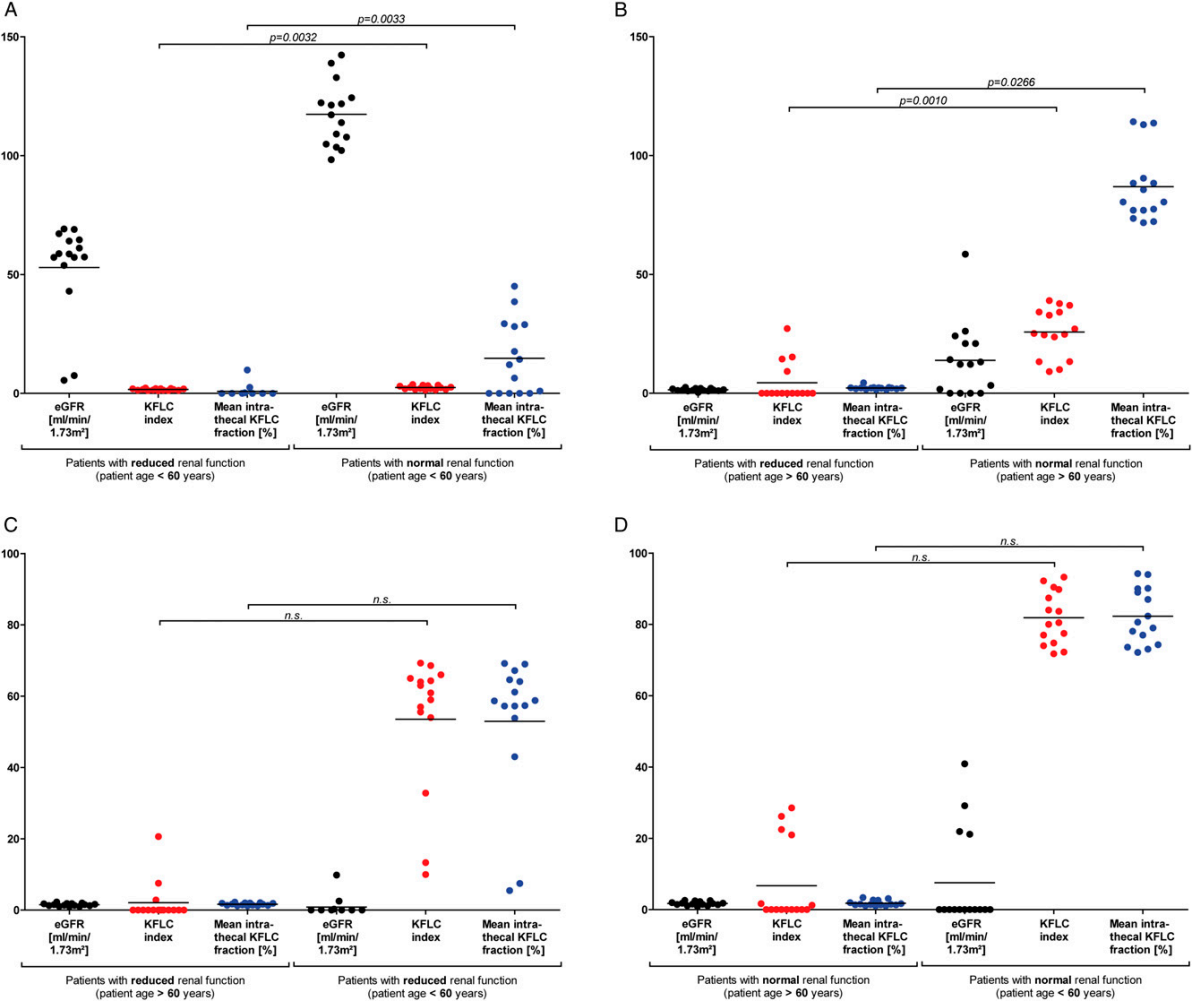
Kappa free light chain (KFLC) index and KFLC fraction in age- and renal function-matched samples. Depicted are comparisons of KFLC indices and intrathecal KFLC fractions in relation to Q_mean_ according to Reiber’s diagram for KFLC (KFLC IF) in samples of “physiological” cerebrospinal fluid (CSF) profile and “non-inflammatory” CSF profile patients. Samples of patients with the most impaired renal function estimated by the glomerular filtration rate (eGFR) according to the CKD-EPI equation (n = 15) were age-matched with samples of patients with the highest possible eGFR (n = 15) (patients below the age of 60 years (A); patients above the age of 60 years (B)). Further, the samples of the oldest patients (n = 15) were eGFR-matched with the youngest possible patient samples (n = 15) (patients with reduced renal function (C); patients with normal renal function (D)). *P*-values are shown above the arrowed line.

Age- and eGFR-matched group comparisons were applied to investigate whether the observed alterations of KFLC index and Reiber’s diagram for KFLC are caused by rising patient age or renal dysfunction (Figure 4). KFLC indices and KFLC IF of patient samples with a “physiological” and “non-inflammatory” CSF profile below and above the age of 60 years with and without renal function impairment (each n = 15) were compared (Figure 4). A comparison of normal and reduced renal function estimated by eGFR in age-matched patient samples revealed significantly lower KFLC indices and KFLC IF in patients with impaired renal function, independently of patient’s age (*P*-values between .0010 and .0266, Figure 4, A and B). In contrast, eGFR-matched comparisons of patient samples with different ages did not reveal significantly different KFLC indices or KFLC IF (Figure 4, C and D). While eGFR-dependent differences of CSF albumin concentrations were not found in age-matched comparisons (Supplementary Material), significantly higher concentrations of albumin were observed in CSF of older patients, when eGFR-matched samples were investigated (*P*-values are .0213 and .0279, Supplementary Material).

## Discussion

This study aimed to investigate the influence of renal function estimated by eGFR according to the CKD-EPI creatinine equation on albumin and KFLC concentrations in serum and CSF. We could identify relevant changes of KFLC and albumin concentrations in relation to impaired renal function, consistent with pathophysiological considerations.

It is known that serum albumin decreases in patients with renal failure. In contrast, serum KFLC accumulates due to missing renal excretion in case of impaired eGFR.^13,14,21^ As both main interpretation methods for KFLC concentrations in CSF, the KFLC index, and KFLC IF depend on the Q_Alb_, the consequences are decreasing KFLC indices and intrathecal KFLC fractions in patient samples of all investigated CSF profiles.

The increasing albumin concentration in CSF in connection with the decreasing renal function though appears to be counterintuitive. We suggest that this is an age-related effect since the CSF flow rate declines and the frequency of renal impairment rises with age.^10^ Furthermore, our results are in line with the literature as the reference value for Q_Alb_ is determined by an age-dependent formula.^10,17,18^ In addition, the comparison of eGFR-matched samples revealed significantly higher CSF albumin concentrations in older patients, while no significant differences were observed in age-matched samples of patients with different renal function.

Although the effect of renal function impairment on CSF protein concentrations is probably age- and CSF flow rate–associated, the influence on the KFLC index and KFLC IF is most likely mediated by altered serum KFLC and albumin concentrations due to renal function impairment. The most likely age-associated effect on CSF albumin is less visible in our “inflammatory” patient samples, probably due to the younger age. In addition, the extent of renal failure is less prominent in this patient group probably explaining the missing association between renal function impairment and lower serum albumin values. As a consequence, the changes of the Q_Alb_ are less pronounced than in the “non-inflammatory” patient samples. In contrast to the “non-inflammatory” patient samples, we did not observe significant changes of KFLC concentrations in CSF in patients with inflammatory neurological diseases depending on renal function or patient age. The reason for the missing dependency might be the clearly higher values of locally produced KFLC in patients with “inflammatory” CSF profiles as shown before in autoinflammatory CNS diseases resulting in a neglectable fraction of diffusion related increase of local KFLC concentration.^22,23^ Due to the reliance of the investigated interpretation methods of KFLC (index and IF) on Q_KFLC_, which decreases with lower renal function, the index and the IF are decreasing with declining renal function in the “inflammatory” patient samples.

In our samples with an “inflammatory” CSF profile, 10% showed an intrathecal KFLC fraction below Q_Kappa_(lim) and 15% presented an index <5.9. The mean eGFR was significantly lower in these patients as compared with patients with an intrathecal KFLC production and eGFR-depending significant differences of KFLC IF and KFLC index in age-matched patient samples were found. Consequently, these patient samples could represent “false” negative KFLC results. However, since 60–65% of the “inflammatory” CSF profile samples with a KFLC index below 5.9 or Reiber’s diagram for KFLC negativity had diagnoses others than autoimmune mediated or infectious processes of the CNS, an intrathecal IgG synthesis is not implicitly to expect, thus these samples might also represent “correct” negative KFLC results. However, a recent study has shown that up to 10% of patients without a neurological disease displayed oligoclonal bands restricted to CSF.^24^ Since a good agreement between the detection of CSF-specific oligoclonal bands and KFLC detection has been demonstrated, pathologic KFLC results might be plausible in the 60–65% of our “inflammatory” CSF profile samples without diagnosis of autoimmune mediated or infectious CNS diseases.^4–9,11,12,22,23,25^ Further, Süße et al reported absolute sensitivity of Reiber’s diagram for KFLC in MS-patients with a normal renal function and normal serum KFLC concentrations; thus, in cases of impaired renal function or otherwise elevated serum KFLC levels, the KFLC IF or index may actually be “false” negative.^12^

Last, qualitative methods in general are less susceptible to confounding factors. Thus, a qualitative method is used for detection of β-trace protein when a false-positive result in the quantitative method is expected.^26^ Apart from its so far assumed higher sensitivity, the qualitative test for oligoclonal bands is not susceptible to spurious alterations of proteins and is thus the method of choice to detect intrathecal IgG synthesis. However, the quantitative detection method of choice constitutes Reiber’s diagram for KFLC since it was shown that the interpretation of KFLC concentrations using a linear index generally leads to more false positive and false negative results, depending on the respective Q_Alb_.^11^ Accordingly, more of our oligoclonal band positive patient samples with an “inflammatory” CSF profile were found to be KFLC positive according to the KFLC IF compared with the KFLC index. Nevertheless, one limitation of Reiber’s quotient diagram is its reliance on a statistical evaluation in comparison with oligoclonal band determination. Another limitation of Reiber’s diagram for KFLC is the lack of confirmatory multicenter studies determining the diagnostic sensitivity in large real-life patient cohorts in the clinical daily routine. To date, some smaller studies applied Reiber’s diagram for KFLC and a great diagnostic sensitivity was achieved.^7,12,25,27–29^ However, it has to be kept in mind that the detection of an intrathecal IgG synthesis by Reiber’s diagram for KFLC, just like oligoclonal band determination is absolutely non-specific for a particular disease.

This study is limited by the usage of daily clinical practice data. Since albumin concentrations in the blood may fluctuate during the day due to variable renal function, the extent of albuminuria may be a better reference for renal damage than creatinine clearance, but such data are not readily available in the daily clinical practice. Furthermore, it is known that the smaller (65 000 D) albumin reaches equilibrium between the CSF and serum compartment within a day after plasmapheresis or immunoadsorption, while the larger IgG (150 000 D) needs up to 3 days.^30^ In view of the low molecular weight of FLC (25 000), equilibration between blood and CSF should occur within a few hours. Since the half-life of KFLC is only 2–6 hours, changes in the blood concentration of KFLC would be readily transferred to the CSF. Therefore, stability of KFLC and albumin concentrations in the blood within a day should be verified to evaluate when a steady-state equilibrium between the CSF and serum compartment is reached, but again such data are not readily available in the daily clinical practice.

## Conclusion

The influence of renal function should be taken into consideration when KFLC is measured in patients suffering from neurological diseases and incongruous results are detected. We suggest using Reiber’s KFLC diagram to determine an intrathecal KFLC fraction rather than the index as the interpretation of KFLC values in quotient diagrams seems to be less susceptible to influence factors like renal function.

## Supplemental Material

sj-pdf-1-cns-10.1177_11795735211042166 – Supplemental Material for The Influence of Renal Function Impairment on Kappa Free Light Chains in Cerebrospinal Fluid Click here for additional data file. Supplemental Material, sj-pdf-1-cns-10.1177_11795735211042166 for The Influence of Renal Function Impairment on Kappa Free Light Chains in Cerebrospinal Fluid by Franz F Konen, Philipp Schwenkenbecher, Ulrich Wurster, Konstantin F Jendretzky, Nora Möhn, Stefan Gingele, Kurt-Wolfram Sühs, Malte J Hannich, Matthias Grothe, Torsten Witte, Martin Stangel, Marie Süße and Thomas Skripuletz in Journal of Central Nervous System Disease

sj-pdf-2-cns-10.1177_11795735211042166 – Supplemental Material for The Influence of Renal Function Impairment on Kappa Free Light Chains in Cerebrospinal Fluid Click here for additional data file. Supplemental Material, sj-pdf-2-cns-10.1177_11795735211042166 for The Influence of Renal Function Impairment on Kappa Free Light Chains in Cerebrospinal Fluid by Franz F Konen, Philipp Schwenkenbecher, Ulrich Wurster, Konstantin F Jendretzky, Nora Möhn, Stefan Gingele, Kurt-Wolfram Sühs, Malte J Hannich, Matthias Grothe, Torsten Witte, Martin Stangel, Marie Süße and Thomas Skripuletz in Journal of Central Nervous System Disease

sj-tif-3-cns-10.1177_11795735211042166 – Supplemental Material for The Influence of Renal Function Impairment on Kappa Free Light Chains in Cerebrospinal Fluid Click here for additional data file. Supplemental Material, sj-tif-3-cns-10.1177_11795735211042166 for The Influence of Renal Function Impairment on Kappa Free Light Chains in Cerebrospinal Fluid by Franz F Konen, Philipp Schwenkenbecher, Ulrich Wurster, Konstantin F Jendretzky, Nora Möhn, Stefan Gingele, Kurt-Wolfram Sühs, Malte J Hannich, Matthias Grothe, Torsten Witte, Martin Stangel, Marie Süße and Thomas Skripuletz in Journal of Central Nervous System Disease

sj-tif-4-cns-10.1177_11795735211042166 – Supplemental Material for The Influence of Renal Function Impairment on Kappa Free Light Chains in Cerebrospinal Fluid Click here for additional data file. Supplemental Material, sj-tif-4-cns-10.1177_11795735211042166 for The Influence of Renal Function Impairment on Kappa Free Light Chains in Cerebrospinal Fluid by Franz F Konen, Philipp Schwenkenbecher, Ulrich Wurster, Konstantin F Jendretzky, Nora Möhn, Stefan Gingele, Kurt-Wolfram Sühs, Malte J Hannich, Matthias Grothe, Torsten Witte, Martin Stangel, Marie Süße and Thomas Skripuletz in Journal of Central Nervous System Disease

sj-tif-5-cns-10.1177_11795735211042166 – Supplemental Material for The Influence of Renal Function Impairment on Kappa Free Light Chains in Cerebrospinal Fluid Click here for additional data file. Supplemental Material, sj-tif-5-cns-10.1177_11795735211042166 for The Influence of Renal Function Impairment on Kappa Free Light Chains in Cerebrospinal Fluid by Franz F Konen, Philipp Schwenkenbecher, Ulrich Wurster, Konstantin F Jendretzky, Nora Möhn, Stefan Gingele, Kurt-Wolfram Sühs, Malte J Hannich, Matthias Grothe, Torsten Witte, Martin Stangel, Marie Süße and Thomas Skripuletz in Journal of Central Nervous System Disease

sj-tif-6-cns-10.1177_11795735211042166 – Supplemental Material for The Influence of Renal Function Impairment on Kappa Free Light Chains in Cerebrospinal Fluid Click here for additional data file. Supplemental Material, sj-tif-6-cns-10.1177_11795735211042166 for The Influence of Renal Function Impairment on Kappa Free Light Chains in Cerebrospinal Fluid by Franz F Konen, Philipp Schwenkenbecher, Ulrich Wurster, Konstantin F Jendretzky, Nora Möhn, Stefan Gingele, Kurt-Wolfram Sühs, Malte J Hannich, Matthias Grothe, Torsten Witte, Martin Stangel, Marie Süße and Thomas Skripuletz in Journal of Central Nervous System Disease
