# Supplementary material for: The Influence of Renal Function Impairment on Kappa Free Light Chains in Cerebrospinal Fluid
Source: J Cent Nerv Syst Dis. 2021 Nov 19;13:11795735211042166. doi: 10.1177/11795735211042166 (PMC8619759; doi:10.1177/11795735211042166)
Supplement: sj-pdf-1-cns-10.1177_11795735211042166 – Supplemental Material for The Influence of Renal Function Impairment on Kappa Free Light Chains in Cerebrospinal Fluid [file sj-pdf-1-cns-10.1177_11795735211042166.pdf]

Supplemental table legend

Supplemental table 1. Diagnoses and kappa free light chain (KFLC) concentrations of all patient sample groups

Shown are diagnosis and KFLC concentrations in cerebrospinal fluid (CSF) and serum of patient samples with "physiological" CSF profile, "non-inflammatory" CSF profile and "inflammatory" CSF profile.

Supplemental figures legend

Supplemental figure 1. Correlation with patient age in "physiological" cerebrospinal fluid (CSF) profile patients

Depicted are the correlations of serum albumin (a1), CSF albumin (a2), serum kappa free light chain (KFLC) (b1) and CSF KFLC (b2) concentrations with patient age in "physiological" CSF profile patients. Further, the correlation between CSF/serum quotients of albumin (a3) and KFLC (b3) and patient age are shown. In c1, the correlation between age and KFLC index (Q_{KFLC} / Q_{Alb}) is depicted, while c2 presents the correlation between age and the intrathecal KFLC fraction in relation to Q_{mean} according to Reiber´s diagram for KFLC (KFLC IF). In d, the correlation of patient age with renal function estimated by the glomerular filtration rate (eGFR) according to the CKD-EPI equation is shown. In the caption, p-values of linear regression and Spearman r (Gaussian distributed values) or Pearson r (nonparametric distributed values) as well as the coefficient of correlation (ρ) are shown.

Supplemental figure 2. Correlation with patient age in "non-inflammatory" cerebrospinal fluid (CSF) profile patients

Depicted are the correlations of serum albumin (a1), cerebrospinal fluid (CSF) albumin (a2), serum kappa free light chain (KFLC) (b1) and CSF KFLC (b2) concentrations with patient age in "non-inflammatory" CSF profile patients. Further, the correlation between CSF/serum quotients of albumin (a3) and KFLC (b3) and patient age are shown. In c1, the correlation between age and KFLC index (Q_{KFLC} / Q_{Alb}) is depicted, while c2 presents the correlation between age and the intrathecal KFLC fraction in relation to Q_{mean} according to Reiber´s diagram for KFLC (KFLC IF). In d, the correlation of patient age with renal function estimated by the glomerular filtration rate (eGFR) according to the CKD-EPI equation is shown. In the caption, p-values of linear regression and Spearman r (Gaussian distributed values) or Pearson r (nonparametric distributed values) as well as the coefficient of correlation (ρ) are shown.

Supplemental figure 3. Correlation with patient age in "inflammatory" cerebrospinal fluid (CSF) profile

Depicted are the correlations of serum albumin (a1), cerebrospinal fluid (CSF) albumin (a2), serum kappa free light chain (KFLC) (b1) and CSF KFLC (b2) concentrations with patient age in "inflammatory" CSF profile patients. Further, the correlation between CSF/serum quotients of albumin (a3) and KFLC (b3) and patient age are shown. In c1, the correlation between age and KFLC index (Q_{KFLC} / Q_{Alb}) is depicted, while c2 presents the correlation between age and the intrathecal KFLC fraction in relation to Q_{mean} according to Reiber´s diagram for KFLC (KFLC IF). In d, the correlation of patient age with renal function estimated by the glomerular filtration rate (eGFR) according to the CKD-EPI equation is shown. In the caption, p-values of linear regression and Spearman r (Gaussian distributed values) or Pearson r (nonparametric distributed values) as well as the coefficient of correlation (ρ) are shown.

Supplemental figure 4. Comparison of cerebrospinal fluid (CSF) albumin concentrations in age- and renal function-matched samples

Depicted are comparisons of cerebrospinal fluid (CSF) albumin concentrations in samples of "physiological" CSF profile and "non-inflammatory" CSF profile patients. Samples of patients with the most impaired renal function estimated by the eGFR according to the CKD-EPI equation (n=15) were age-matched with samples of patients with the highest eGFR (n=15) (patients below the age of 60 years (a); patients above the age of 60 years (b)). Further, the samples of the oldest patients (n=15) were eGFR-matched with the youngest patient samples (n=15) (patients with reduced renal function (c); patients with normal renal function (d)). P-values are shown above the arrowed line.
