# Supplementary material for: The Influence of Renal Function Impairment on Kappa Free Light Chains in Cerebrospinal Fluid
Source: J Cent Nerv Syst Dis. 2021 Nov 19;13:11795735211042166. doi: 10.1177/11795735211042166 (PMC8619759; doi:10.1177/11795735211042166)
Supplement: sj-pdf-2-cns-10.1177_11795735211042166 – Supplemental Material for The Influence of Renal Function Impairment on Kappa Free Light Chains in Cerebrospinal Fluid [file sj-pdf-2-cns-10.1177_11795735211042166.pdf]

Diagnosis	Physiological CSF profile (n=139)	Non-inflammatory CSF profile (n=146)	Inflammatory CSF profile (n=172)
Autoimmune mediated or infectious processes in CNS, n (%)	0	0	154 (90%)
Serum KFLC concentration of "autoimmune mediated or infectious processes in CNS" (mg/l), mean (min-max)	0	0	11.7 (2-43.2)
CSF KFLC concentration of "autoimmune mediated or infectious processes in CNS" (mg/l), mean (min-max)	0	0	5 (0.1-35.2)
Headache, diffuse paresthesia or psychiatric diagnosis, n (%)	39 (28%)	27 (19%)	3 (2%)
Serum KFLC concentration of "headache, diffuse paresthesia or psychiatric diagnosis" (mg/l), mean (min-max)	12.4 (7.8-34.8)	14.6 (7.3-53.2)	8.9 (6.4-11.8)
CSF KFLC concentration of "headache, diffuse paresthesia or psychiatric diagnosis" (mg/l), mean (min-max)	0.12 (0.04-0.4)	0.22 (0.05-0.6)	0.1 (0.1)
Peripheral nerve pathologies, n (%)	39 (28%)	41 (28%)	7 (4%)
Serum KFLC concentration of "peripheral nerve pathologies" (mg/l), mean (min-max)	13.5 (5.6-29)	19.3 (8.1-95.2)	13.3 (9.4-19.7)
CSF KFLC concentration of "peripheral nerve pathologies" (mg/l), mean (min-max)	0.14 (0.07-0.4)	0.31 (0.08-1.4)	0.64 (0.1-1.9)
Epilepsy, n (%)	22 (16%)	27 (19%)	2 (1%)
Serum KFLC concentration of"epilepsy" (mg/l), mean (min-max)	20 (6.4-90.5)	17 (7-50.3)	5.4 (4-6.7)
CSF KFLC concentration of"epilepsy" (mg/l), mean (min-max)	0.2 (0.03-0.7)	0.27 (0.1-1.2)	0.1 (0.1)
Vascular diseases of the CNS, n (%)	17 (12%)	18 (12%)	6 (3%)
Serum KFLC concentration of "vascular diseases of the CNS" (mg/l), mean (min-max)	15.6 (8.2-65.4)	16.3 (7.6-45.4)	19.2 (8.5-42.4)
CSF KFLC concentration of "vascular diseases of the CNS" (mg/l), mean (min-max)	0.17 (0.1-0.4)	0.27 (0.1-0.7)	1.07 (0.11-3.88)
Neuro-degenerative diseases, n (%)	16 (12%)	12 (8%)	0
Serum KFLC concentrations of "neuro-degenerative diseases" (mg/l), mean (min-max)	17.5 (8-31.5)	22.7 (7.7-39)	0
CSF KFLC concentrations of "neuro-degenerative diseases" (mg/l), mean (min-max)	0.18 (0.08-0.45)	0.32 (0.06-0.69)	0
Idiopathic intracranial hypertension (IIH) or normal pressure hydrocephalus (NPH), n (%)	6 (4%)	3 (2%)	0
Serum KFLC concentration of "idiopathic intracranial hypertension (IIH) or normal pressure hydrocephalus (NPH)" (mg/l), mean (min-max)	18.1 (9.5-26.9)	15.5 (11.7-18.8)	0
CSF KFLC concentration of "idiopathic intracranial hypertension (IIH) or normal pressure hydrocephalus (NPH)" (mg/l), mean (min-max)	0.12 (0.09-0.14)	0.18 (0.09-0.31)	0
Encephalopathy, n (%)	0	15 (10%)	0
Serum KFLC concentration of "encephalopathy" (mg/l), mean (minx-max)	0	14.6 (7.1-32.5)	0
CSF KFLC concentration of "encephalopathy" (mg/l), mean (minx-max)	0	0.24 (0.03-0.55)	0
Malignant diseases of the CNS, n (%)	0	3 (2%)	0
Serum KFLC concentration of "malignant diseases of the CNS" (mg/l), mean (min-max)	0	54.1 (12.4-130)	0
CSF KFLC concentration of "malignant diseases of the CNS" (mg/l), mean (min-max)	0	0.9 (0.4-1.9)	0

Supplemental table 1
