# Supplementary figures and images for: The Influence of Renal Function Impairment on Kappa Free Light Chains in Cerebrospinal Fluid
Source: J Cent Nerv Syst Dis. 2021 Nov 19;13:11795735211042166. doi: 10.1177/11795735211042166 (PMC8619759; doi:10.1177/11795735211042166)

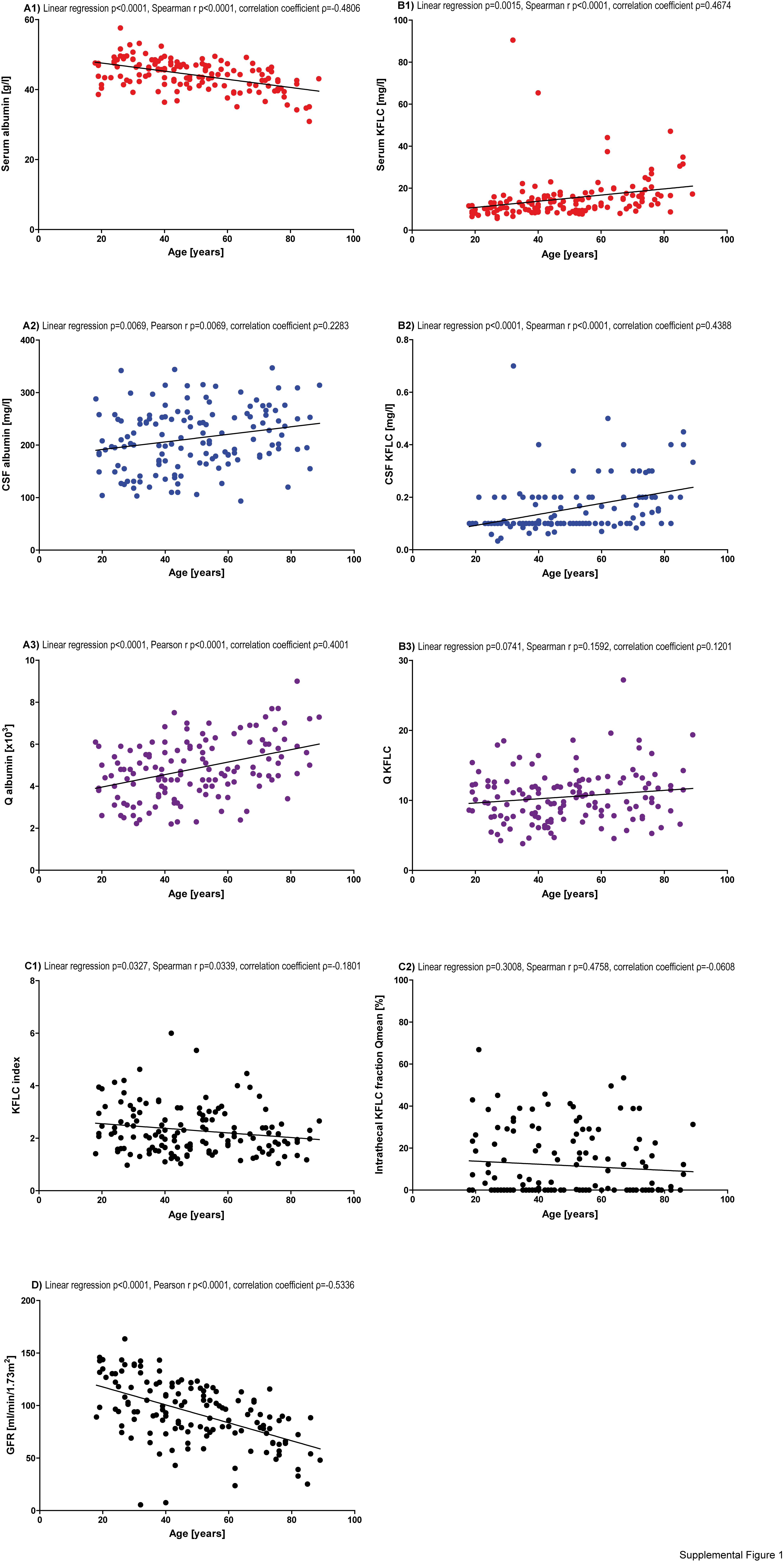

Supplement: sj-tif-3-cns-10.1177_11795735211042166 – Supplemental Material for The Influence of Renal Function Impairment on Kappa Free Light Chains in Cerebrospinal Fluid [file sj-tif-3-cns-10.1177_11795735211042166.tif]

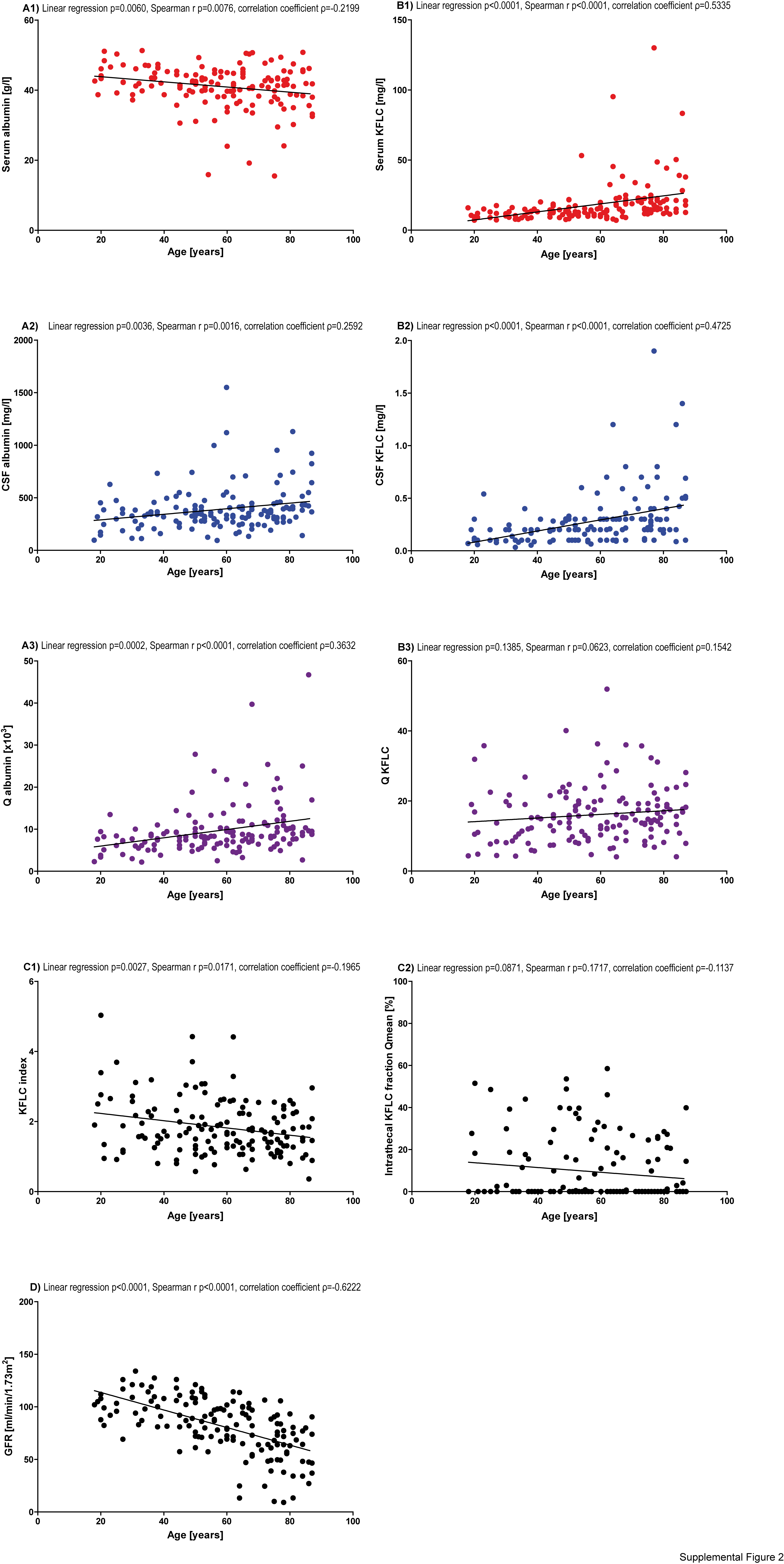

Supplement: sj-tif-4-cns-10.1177_11795735211042166 – Supplemental Material for The Influence of Renal Function Impairment on Kappa Free Light Chains in Cerebrospinal Fluid [file sj-tif-4-cns-10.1177_11795735211042166.tif]

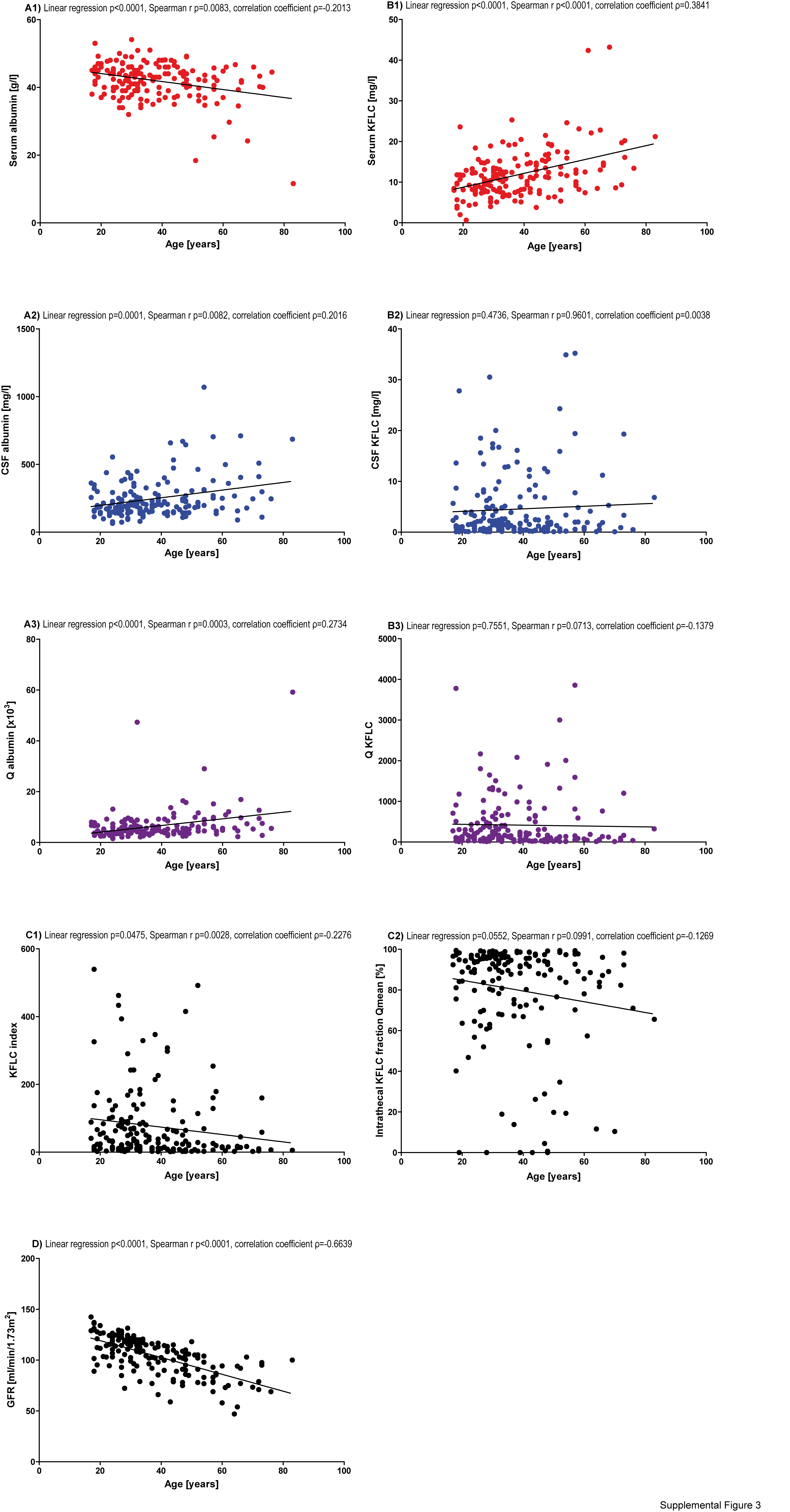

Supplement: sj-tif-5-cns-10.1177_11795735211042166 – Supplemental Material for The Influence of Renal Function Impairment on Kappa Free Light Chains in Cerebrospinal Fluid [file sj-tif-5-cns-10.1177_11795735211042166.tif]

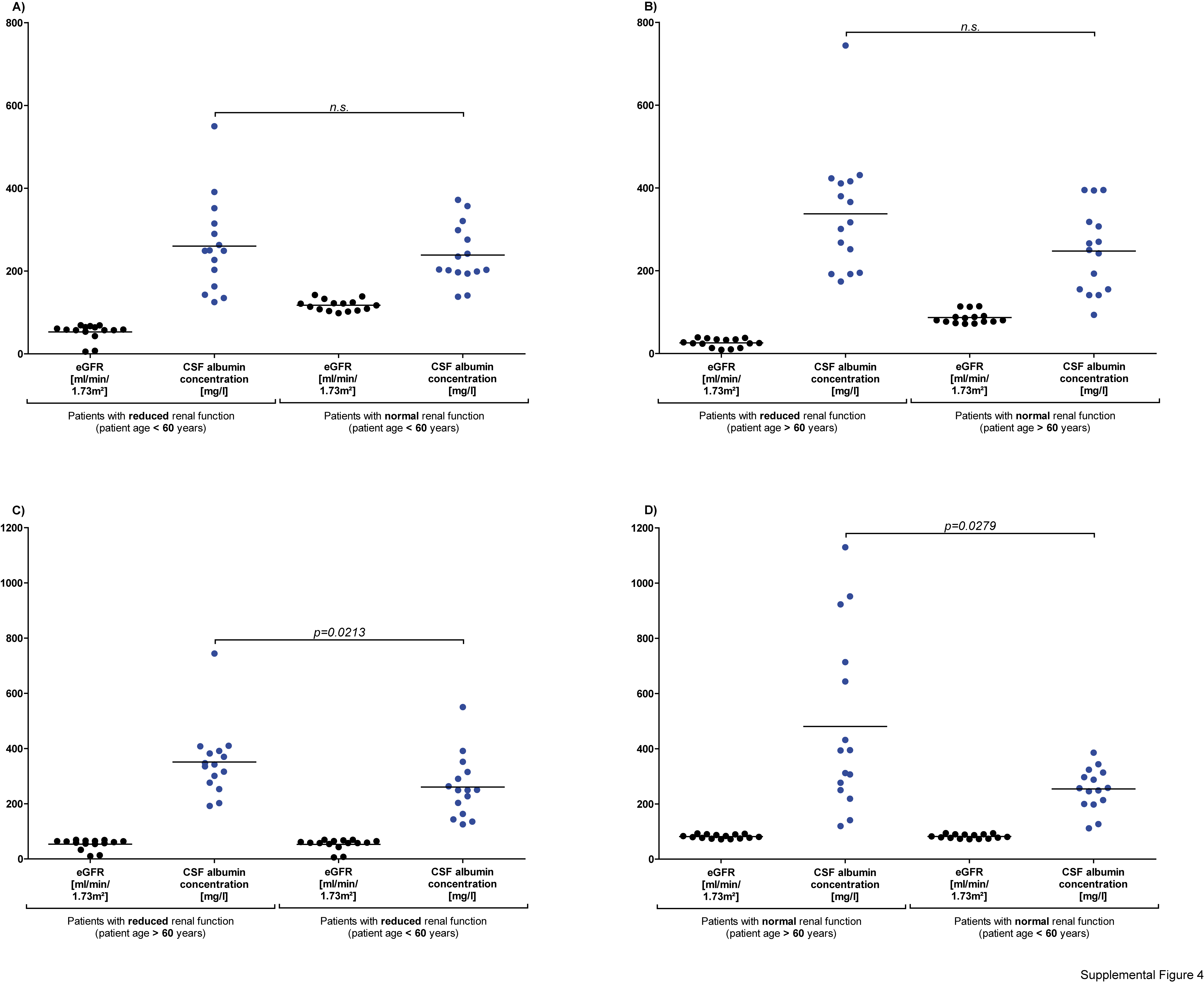

Supplement: sj-tif-6-cns-10.1177_11795735211042166 – Supplemental Material for The Influence of Renal Function Impairment on Kappa Free Light Chains in Cerebrospinal Fluid [file sj-tif-6-cns-10.1177_11795735211042166.tif]
